# Neurologic, clinical, and immunologic features in a cohort of HTLV-1 carriers with high proviral loads

**DOI:** 10.1007/s13365-020-00847-y

**Published:** 2020-05-08

**Authors:** Sheila N. Ferraz, Gabriela F. Costa, José Abraão Carneiro Neto, Thiago Hebert, Cassius J. V. de Oliveira, Mariele Guerra, Lívia M. A. Oliveira, Edgar M. Carvalho

**Affiliations:** 1 Immunology Service, Professor Edgard Santos University Hospital, Federal University of Bahia, Salvador, Brazil; 2 Escola Bahiana de Medicina e Saúde Pública, Salvador, Bahia Brazil; 3 Instituto Gonçalo Moniz (FIOCRUZ-BA), Fundação Oswaldo Cruz, Rua Waldemar Falcão, 121, Candeal, Salvador, Bahia Brazil; 4 National Institute of Science and Technology in Tropical Diseases (INCT-DT), CNPq, Salvador, Brazil

**Keywords:** HTLV-1-associated myelopathy, HTLV-1, Proviral load, HTLV-1 carriers, Myelopathy

## Abstract

A high proviral load (PVL) is recognized as a risk factor for human T cell leukemia virus-1-associated myelopathy/tropical spastic paraparesis (HAM/TSP), but there is a lack of prospective studies evaluating whether or not HTLV-1 carriers with high PVL are at risk of developing HAM/TSP or other HTLV-1-related diseases. Here, we compare the incidence of clinical manifestations and the cytokine levels in 30 HTLV-1 carriers with high (> 50,000 copies/10^6^ PBMC) and an equal number of subjects with low proviral load. Participants were followed for 3 to 16 years (median of 11 years). The PVL, IFN-γ, TNF, and IL-10 levels were quantified at entry and at the end of the follow-up. Among the self-reported symptoms in the initial evaluation, only the presence of paresthesia on the hands was more frequent in the group with high PVL (*p* < 0.04). The production of IFN-γ was higher in the group with high PVL group (median of 1308 versus 686 pg/ml, *p* < 0.011) when compared with the control group in the first assessment. There was no difference in the occurrence of urinary symptoms or erectile dysfunction, periodontal disease, Sicca syndrome, and neurologic signs between the two groups during the follow-up. The observation that none of the HTLV-1 carriers with high PVL and with exaggerated inflammatory response progressed to HAM/TSP indicates that other factors in addition to the PVL and an exaggerated immune response are involved in the pathogenesis of HAM/TSP.

## Introduction

The human T cell leukemia virus-1 (HTLV-1) was the first human retrovirus to be described (Poiesz et al. 1980) and is the etiologic agent of adult T cell leukemia/lymphoma (ATL) (Uchiyama et al. 1977) and HTLV-1-associated myelopathy or tropical spastic paraparesis (HAM/TSP) (Osame et al. 1986; Gessain et al. 1985). The HTLV-1 virus preferentially infects CD4 T cells but is also found in CD8 T cells, B cells, and dendritic cells (Bangham 2018; Martin et al. 2016). HTLV-1 infection activates genes that induce T cell proliferation and activation with an exaggerated production of proinflammatory cytokines such as TNF, IL-1, IFN-γ, TGF-β, CXCL-9, and CXCL-10 (Ando et al. 2013; Futsch et al. 2017; Sato et al. 2013). The high proviral load usually above 50,000 copies per 10^6^ cells and the exacerbated inflammatory response are considered the main biomarkers of HAM/TSP (Grassi et al. 2011; Olindo et al. 2006; Santos et al. 2012). HAM/TSP is characterized by a slowly progressive spastic paraparesis, neurogenic bladder, and less visible sensory signals (Araújo et al. 2009). About 20% of individuals infected with HTLV-1 without HAM/TSP have urinary dysfunctions, especially due to overactive bladder (Troisgros et al. 2017). Erectile dysfunction is also observed in more than 40% of virus-infected subjects (de Oliveira et al. 2017). In addition to the aforementioned diseases, individuals infected with HTLV-1 have more chronic periodontitis, Sicca syndrome, and arthropathy than seronegative individuals (Motokawa et al. 1996; Poetker et al. 2011; Caskey et al. 2008).

Previous studies with HTLV-1-infected subjects who did not fulfill the criteria for HAM/TSP have shown a greater occurrence of neurologic symptoms, such as weakness in the lower limbs, hyperreflexia, and altered vibratory sensitivity, as well as urinary dysfunction and erectile dysfunction, than in seronegative controls (Biswas et al. 2009; Caskey et al. 2008). It has also been observed that many HTLV-1-infected individuals who do not meet the criteria for HAM/TSP present neurological complaints related to sensorial, motor, urinary, or autonomic manifestations (Tanajura et al. 2015). Usually in virus-infected individuals who exhibit these symptoms or diseases, the PVL is high or virus proteins are documented in the compromised tissue, supporting the important role of PVL in the passage of viruses to the tissues as a risk factor for carriers’ progress to disease. However, a significant proportion of HTLV-1 carriers have PVL as high as patients with HAM/TSP (Santos et al. 2006; Martins et al. 2017). The majority of the studies correlating viral load and immunologic responses with clinical manifestations in HTLV-1 are cross-sectional. There is a lack of prospective studies evaluating if subjects with high viral burdens for long periods of time will develop HTLV-1-associated diseases as well as immunological abnormalities similar to those observed in patients with HAM/TSP.

## Methods

### Type of study and participants

This is a prospective cohort study with the participation of HTLV-1-infected subjects admitted between 2001 and 2016 in the Multidisciplinary HTLV-1 Clinic of the University Hospital Professor Edgard Santos, Salvador, Bahia, Brazil. The study subjects were classified as exposed (high PVL) or non-exposed (low PVL). The exposed group had PVL greater than 50,000 copies/10^6^ PBMC (Grassi et al. 2011) without any evidence of probable or definitive myelopathy (De Castro-costa et al. 2006). Non-exposed subjects were defined as HTLV-1 carriers with PVL lower than 50,000 copies/10^6^ cells at admission.

The HTLV-1 infection was diagnosed by the enzyme-linked immunosorbent assay (ELISA)—(Cambridge Biotech Corp., Worcester, MA, USA) and confirmed by Western blot test, HTLV blot (Genelabs, Singapore). Initially, 92 HTLV-1 carriers were identified, and 31 had high PVL. As one of these patients had neurosyphilis, he was excluded. Thus, 30 subjects with a high PVL and 30 with a low PVL matched for age (± 5 years) and gender participated in the study. None of the subjects was taking immunosuppressive drugs or was using phosphodiesterase inhibitors.

### Study design

The subjects answered a standard questionnaire and had a clinical and neurological physical examination performed once a year. Information about how they were referred to the clinic, previous history of blood transfusions, breast feeding, and sexual behavior were recorded. Patients were evaluated by neurologists, urologists, rheumatologists, dentists, and psychologists. The outcomes determined were Sicca syndrome, periodontitis, urinary dysfunction, erectile dysfunction, walking difficulty, running difficulty, hyperreflexia (grade 3 or 4), and Babinski reflexes. Sicca syndrome was defined by xerophthalmia, and evidence of dry mouth and chronic periodontal diseases by the criteria was established by the International Association of Periodontal Disease. Low urinary tract symptoms such as nocturia, frequency, urgency, and incontinence were recorded, as well as void dysfunction. The erectile dysfunction was determined by the international index of erectile function (IIEF-5).

### Cell separation, cell culture, and determination of cytokine

Peripheral blood mononuclear cells (PBMCs) were isolated from heparinized blood samples by density gradient centrifugation with Ficoll-Hypaque (GE Healthcare Bio-Sciences, Uppsala, Sweden). The cells were cultured in RPMI 1640 (Life Technologies Gibco BRL, Grand Island, NY), 10% human AB serum (Sigma, St. Louis, MO, USA), glutamine, HEPES, and antibiotics (complete RPMI). Cytokines were determined in supernatants of unstimulated PBMC cultures as previously described (Santos et al. 2012). Briefly, after mononuclear cell separation, the cells were washed twice in saline and were adjusted to the concentration of 3 × 10^6^ cells/ml in RPMI 1640 (Life Technologies Gibco BRL, Gran Island, NY, USA) supplemented with 10% of fetal bovine serum (Sigma, St Louis, MO, USA), glutamine, HEPES, and antibiotics. Unstimulated cells, 3 × 10^6^ cells/ml, were incubated for 72 h at 37 °C 5% CO_2_, and the supernatants were harvested. Cytokines were determined in supernatants of unstimulated PBMC cultures by sandwich ELISA using reagents from BD Biosciences Pharmingen, San Jose, CA, USA, and the results are expressed in pg/ml.

### Determination of the Proviral load

The DNA was extracted from 10^6^ PBMCs using proteinase K and salting-out method. The HTLV-1 proviral load was quantified using a real-time Taq-Man PCR method as previously described using the ABI Prism 7700 Sequence detector system (Applied Biosystems) (Dehée et al. 2002). Albumin DNA was used as an endogenous reference. The normalized value of the HTLV-1 proviral load was calculated as the ratio of HTLV-1 DNA average copy number/albumin DNA average copy number × 2 × 10^6^ and expressed as the number of HTLV-1 copies per 10^6^ PBMCs.

### Statistical analysis

All the data were kept in an electronic data bank, the Research Electronic Data Capture (Harris et al. 2009). Data was expressed as median and interquartile (IQ) range in all figures. Categorical variables were analyzed using the Pearson’s Chi-squared test or Fisher exact test. The proviral load and cytokine levels were expressed in median, and the comparison was performed by the Mann-Whitney *U* test. For the paired samples, we used the McNemar test. The logistic regression analysis was used to identify independent variables associated with the existence of mono/oligosymptomatic subjects. We also determined the intraindividual PVL and cytokine changes by the coefficient of variation (Martins et al. 2017). Correlations between two variables were examined by Spearman rank correlation analysis. All statistical analysis was performed using SPSS version 17 and were considered significant when *p* < 0.05.

## Results

A total of 60 individuals infected by HTLV-1 were eligible for this study and included in the analysis. The demographic, epidemiological, and clinical features of the participants are shown in Table 1. There was a predominance of female individuals (66.7%). These subjects were mainly referred by blood banks, being 60% of the total in the group of subjects with high proviral load and 80% in subjects with low proviral load. The mean age was slightly higher in the group of non-exposed subjects, 56.2 years versus 55.5 years (*p* = 0.81). In the group of carriers with high PVL, the HTLV-1 carriers were followed a median of 11 years versus 11.5 years in the non-exposed group (*p* = 0.57).

**Table 1:** Demographic, epidemiologic characteristics, and clinical aspects of HTLV-1 carriers, classified according to the proviral load at study entry

Characteristic	High proviral load carriers (*n* 30)	Low proviral load carriers (*n* 30)	*p* value
Female sex	20 (66.7%)	20 (66.7%)	
Median age. y	55.5 (± 11.6)	56.2 (± 11.2)	= 0.81^c^
Patient Referral			= 0.19^a^
Blood banks	18 (60%)	24 (80%)	
Other clinics	1 (3.3%)	0	
Relatives	1 (3.3%)	2 (6.7%)	
Others	10 (33.3%)	4 (13.3%)	
Blood transfusion	4 (13.8%)	1 (3.3%)	= 0.19^b^
Years of follow-up (median [interquartile range])	11 (8.5–13)	11.5 (8–12)	=0 .57^c^
Comorbidities			
Diabetes mellitus	4 (13.3%)	3 (10%)	> 0.99^b^
Hypothyroidism	3 (10%)	1 (3.3%)	= 0.61^b^
Osteoarthritis	7 (23.3%)	13 (43.3%)	= 0.10^a^
HBV infection	0	1(3.3%)	> 0.99^b^
HCV infection	0	1 (3.3%)	> 0.99^b^

*Chi-square or Fisher’s exact test was used if characteristic was categorical and Mann-Whitney *U* test if continuous

a χ2 test; b Fisher’s exact test; c Mann-Whitney *U* test

HTLV-1, human T-cell leukemia virus-1; HBV, hepatitis B virus; HCV, hepatitis C virus

**Fig. 1:**
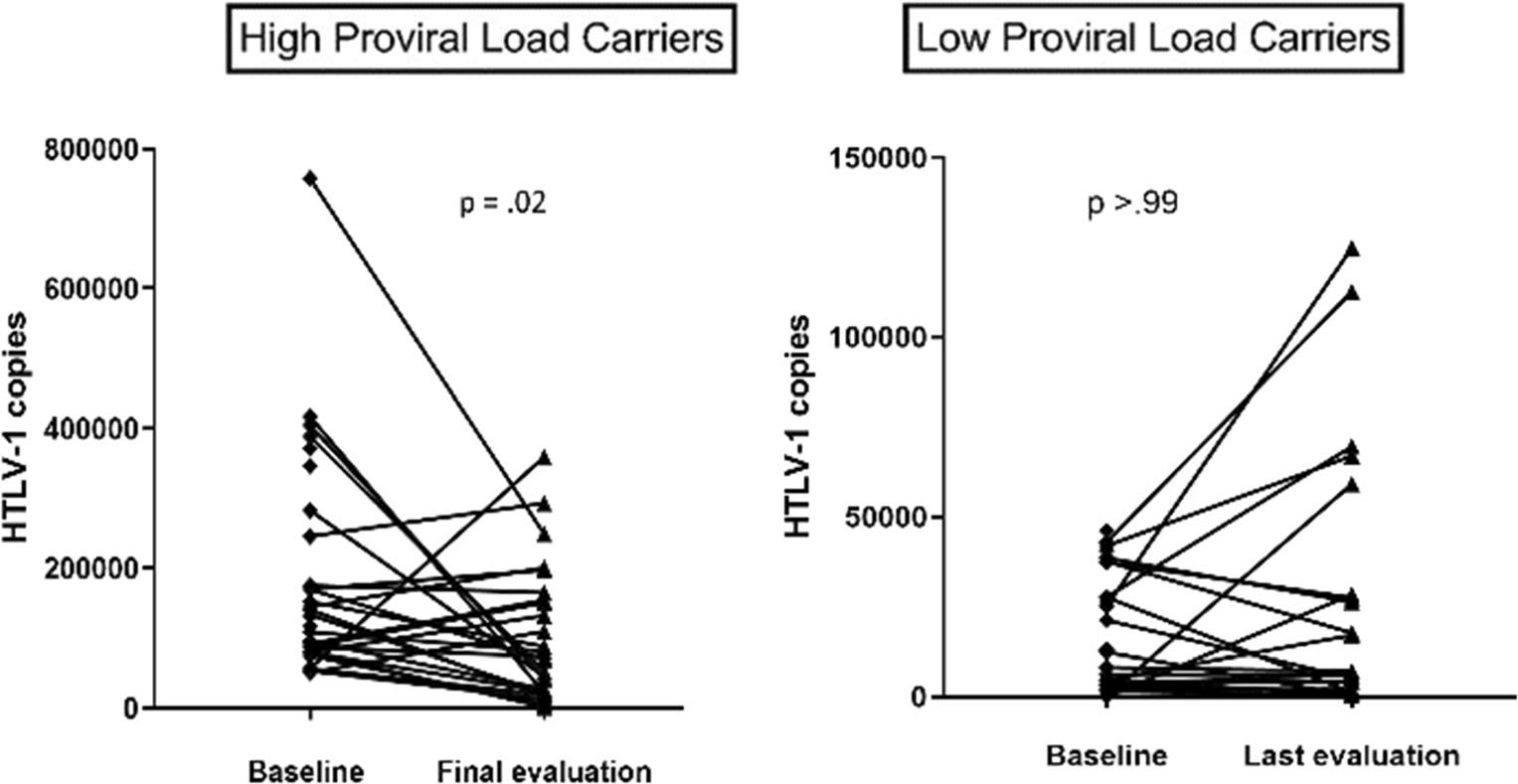
Comparative analysis between the baseline proviral load and the last evaluation in HTLV-1 carriers with high and low proviral load. Data represents the proviral load expressed as number of HTLV-1 copies per 10^6^ peripheral blood mononuclear cells. Wilcoxon signed-rank test was used for the statistical analysis. *p* value < 0.05 was considered significant

The comparative analysis of HTLV-1 carriers at study entry and in the last evaluation is shown in Table 2. No significant differences were identified between the neurological signs and symptoms at the final follow-up in the exposed group indicating that carriers with high proviral load were not more likely than controls to report complaints about the ability to run and to walk with urinary tract symptoms and paresthesia in the feet. There was also no difference in the frequency of joint pain and periodontal disease during the follow-up. The cases had more xerostomia at the last evaluation compared with the entry. Moreover, in a logistic regression analysis, there was no association between PVL and cytokine level with mono/oligosymptomatic subjects (data not shown). In summary none of the HTLV-1 carriers during the follow-up developed probable HAM or definitive HAM/TSP. The evaluation of the decrease in PVL between the two groups at the entry and during the follow-up is shown in Fig. 1. While there was a decrease in the high PVL among carriers during the follow-up, there was no significant change in PVL among the controls. However, when the coefficient of variation was evaluated, there was no difference in the PVL during the follow-up.

**Table 2:** Clinical symptoms and signs in HTLV-1-infected subjects with high or low proviral load at admission and in the last evaluation

Clinical features	Low proviral load carriers			High proviral load carriers			Comparison between groups	
	Admission	Final evaluation	*p* value^a^	Admission	Final evaluation	*p* value^a^	Admission *p* value	*Final evaluation p* value
Urological symptoms	4 (13.3%)	4 (13.3%)	> 0.99	5 (16.7%)	5 (16.7%)	1	> 0.99^b^	0.99^b^
Effort urinating	3 (10%)	0	0.25	1 (3.3%)	2 (6.7%)	> 0.99	0.61^b^	0.49^b^
Urgency	1 (3.3%)	0	> 0.99	0	0	–	> 0.99^b^	–
Stress urinary incontinence	0	0	–	3 (10%)	1 (3.3%)	0.63	0.24^b^	> 0.99^b^
Erectile dysfunction	0	3/10 (30%)	0.25	0	0	–	–	0.21^b^
Others	0	1 (3.3%)	> 0.99	1 (3.3%)	2 (6.7%)	> 0.99	> 0.99^b^	> 0.99^b^
Neurological symptoms								
Difficulty running	2 (6.7%)	6 (20%)	0.22	3 (10%)	5 (16.7%)	0.69	> 0.99^b^	0.74^c^
Difficulty walking	0	3 (10%)	0.25	1 (3.3%)	2 (6.7%)	> 0.99	> 0.99^b^	> 0.99^b^
Hand numbness	2 (6.7%)	4 (13.3%)	0.68	8 (26.7%)	5 (16.7%)	0.51	0.04^c^	> 0.99^b^
Foot numbness	2 (6.7%)	4 (13.3%)	0.68	7 (23.3%)	6 (20%)	> 0.99	0.14^b^	0.49^c^
Biceps hyperreflexia	4 (13.3%)	2 (6.7%)	0.5	1 (3.3%)	1 (3.3%)	1	0.35 ^c^	> 0.99^b^
Patellar hyperreflexia	4 (13.3%)	5 (16.7%)	> 0.99	5(16.7%)	7(23.3%)	0.62	> 0.99^b^	0.52^c^
Babinski sign	0	0		0	0			
Rheumatological symptoms								
Joint pain								
Monoarticular	1/28 (3.6%)	4 (13.3%)	0.5	2 (6.7%)	5 (16.7%)	0.37	> 0.99^b^	> 0.99^b^
Oligoarticular	1/28 (3.6%)	3 (10%)	0.62	1 (3.3%)	7 (23.3%)	0.07	> 0.99^b^	0.16^c^
Polyarticular	0/28	2 (6.7%)	0.5	0	1 (3.3%)	> 0.99	–	> 0.99^b^
Xerostomia	4 (13.3%)	6/23 (26.1%)	0.68	3 (10%)	9/25 (36%)	0.03	> 0.99^b^	0.46^c^
Xerophthalmia	6/29 (20.7%)	6 (20%)	> 0.99	5 (16.7%)	13 (43.3%)	0.06	0.69^c^	0.052^c^
Periodontal disease	6/16 (37.5%)	12/21 (57.1%)	> 0.99	11/24 (45.8%)	12/24 (50%)	> 0.99	0.60^c^	0.63^c^

*Chi-square or Fisher’s exact test was used if characteristic was categorical and McNemar test for paired data

a McNemar test; B Fisher’s exact test; c χ^2^ Test

**Fig. 2:**
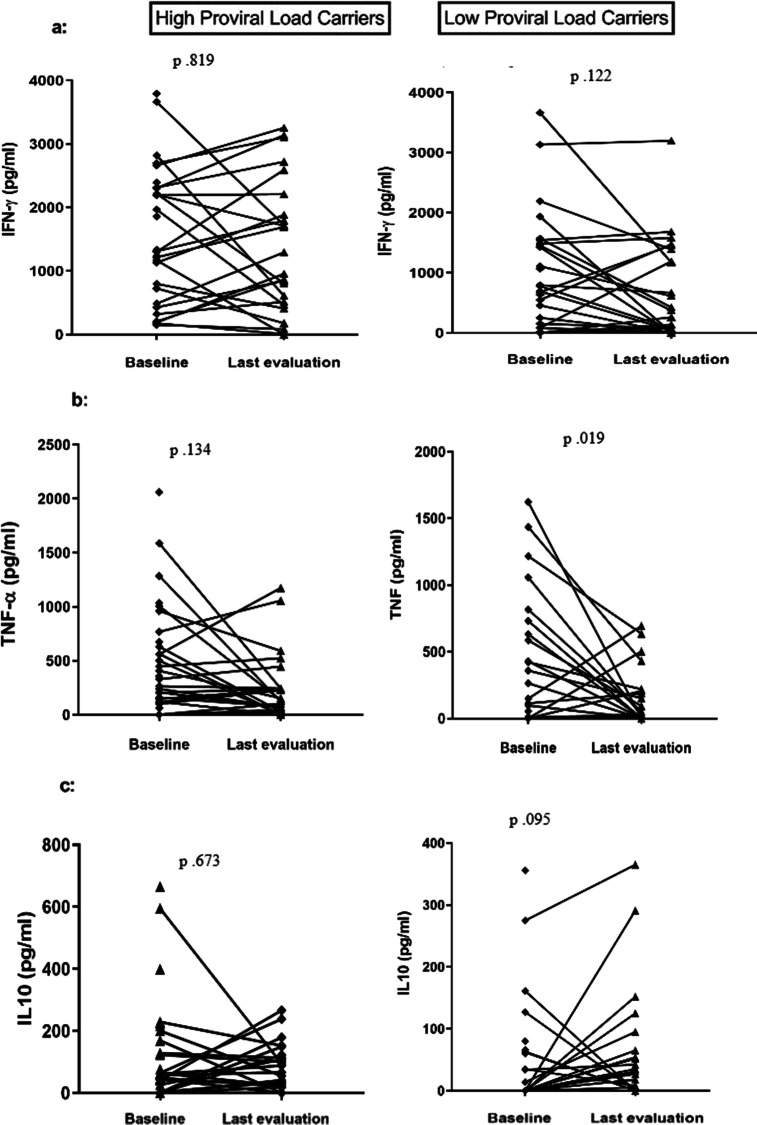
Cytokine concentrations in supernatants of unstimulated mononuclear cell cultures of HTLV-1 carriers with high and low proviral load at study entry and at the last evaluation: **a** IFN-γ, **b** TNF, **c** IL10. Cytokine levels were measured by ELISA in supernatant of unstimulated 3 × 10^6^ peripheral blood mononuclear cells after 72 h of incubation. Wilcoxon signed-rank test was used for the statistical analysis. *p* value < 0.05 was considered significant

The cytokine production at baseline and in the last evaluation is shown on Fig. 2. The concentration of IFN-γ in supernatants of PBMC was higher in the group with a high proviral load (median of 1308 versus 686 pg/ml, *p* = 0.011) when compared with the control group in the first evaluation, as well as the concentration of IL-10 (median of 53 pg/ml versus 0 pg/ml, *p* = 0.01). There were no differences in the levels of TNF and IL-10 at baseline, and in the last evaluation, there was a decrease in TNF concentration in both groups, with a significant difference in the group with low proviral load with median of 105 pg/ml at admission versus 22 pg/ml in the last evaluation, *p* = 0.01).

The correlations between the production of cytokines in supernatants of PBMC as well as between cytokines and PVL are shown in Table 3. There was a direct correlation between IFN-γ levels and PVL in low PVL carriers in the first (*r* = 0.55) and in the last (*r* = 0.65) evaluation and between TNF and PVL in the same group in the last evaluation (*r* = 0.43). Regarding the correlation between proinflammatory versus anti-inflammatory cytokines, it is important that the only strong correlation observed was a direct correlation between IFN-γ and IL-10 in the high PVL (*r* = 0.76) in the last evaluation. There was a direct correlation between TNF and IL-10 in all evaluations except in the first evaluation of the group with low PVL. Regarding the proinflammatory cytokines, there was a direct correlation between IFN-γ and TNF in all evaluations in both groups.

**Table 3:** Correlations between serum cytokines and proviral load from HTLV-1 carriers at study entry and at the last evaluation

Correlations	High proviral load carriers		Low proviral load carriers	
	Study entry	Last evaluation	Study entry	Last evaluation
IFN-γ x PVL	− 0.013 (= 0.95)	− 0.23 (= 0.29)	0.55 (< 0.01)	0.65 (< 0.001)
TNF x PVL	0.117 (= 0.54)	0.002 (= 0.99)	0.29 (= 0.12)	0.43 (= 0.03)
IL10 x PVL	0.15 (= 0.41)	0.27 (= 0.24)	0.30 (= 0.11)	0.20 (= 0.37)
IFN-γ x TNF	0.45 (= 0.01)	0.72 (< 0.001)	0.54 (< 0.001)	0.60 (= 0.001)
IFN-γ x IL10	0.30 (= 0.10)	0.76 (< 0.001)	0.45 (= 0.01)	0.31 (= 0.14)
TNF x IL10	0.48 (< 0.01)	0.45 (= 0.03)	0.35 (= 0.06)	0.45 (= 0.03)

Spearman correlations were calculated between parameters. The “r” indexes are shown, as well the (*p* value)

PVL, proviral load

## Discussion

The HAM/TSP only occurs in less than 5% of HTLV-1-infected subjects (Gessain and Mahieux 2012), but there are other clinical manifestations associated with this virus such as ATL (Poiesz et al. 1980), Sicca syndrome (Lima et al. 2016), periodontal disease (Garlet et al. 2010), and arthropathy (Frenzel et al. 2014). Moreover, 40% of the HTLV-1-infected subjects without HAM/TSP have neurologic symptoms and signs related to spinal cord involvement, such as paresthesia, weakness in the inferior limbs, difficult walking, urologic manifestations of neurogenic bladder, and erectile dysfunction (Tanajura et al. 2015; Biswas et al. 2009; Poetker et al. 2011; Caskey et al. 2008). The HTLV-1 PVL has been considered the most important biomarker of HAM/TSP, and patients with HAM/TSP have higher production of proinflammatory cytokines than HTLV-1 carriers (Domingos et al. 2017; Guerreiro et al. 2006; Santos et al. 2012). It is known that HTLV-1 carriers may present PVL and proinflammatory cytokine levels as high as what is observed in HAM/TSP (Santos et al. 2004). However, there is a lack of longitudinal studies evaluating if overtime carriers with high HTLV-1 PVL will develop more clinical and neurogenic diseases than carriers with low proviral load or if they are at a high risk to develop HAM/TSP. In the present study, we showed in a cohort study that HTLV-1 carriers who have a high viral burden and also produce high levels of proinflammatory cytokines may not develop more clinical and neurological signs and symptoms than HTLV-1 carriers with low PVL.

The two groups of HLV-1 carriers of the present study had similar demographic and epidemiologic profiles at admission, but they differ regarding the proviral load and the production of proinflammatory cytokines. Additionally, carriers from both groups had no difference in the clinical or neurological examination at admission. The only exception was the presence of paresthesia on the hands, which was more frequent in the group with a high PVL. It is relevant that two previous studies aimed to identify clinical and neurological manifestations related to HTLV-1 found higher frequencies of Sicca syndrome, periodontal disease, urinary complaints of neurogenic bladder, hyperreflexia, and even difficulty walking in HTLV-1 carriers than in the no infected subjects (Caskey et al. 2008; Poetker et al. 2011). The higher frequency of clinical and neurological manifestations in carriers in these previous studies compared with our data may be explained by the improvement in knowledge about the clinical spectrum of HTLV-1 infection in the last 10 years, mainly due to the understanding that urinary dysfunction was one of the manifestation associated to HTLV-1 infection. In the present study, HTLV-1 carriers were defined as patients who did not have HAM/TSP or probable HAM/TSP, that is, a form of HTLV-1 infection characterized mainly by urinary dysfunction (De Castro-costa et al. 2006; Tanajura et al. 2015).

The enhanced T cell activation contributes to the passage of cells through the brain-blood barrier and persistent secretion of proinflammatory cytokines (Bangham 2018). A previous study showed that a proviral load higher than 50,000 copies for 10^6^/lymphocytes is a good predictor for the diagnosis of HAM/TSP (Grassi et al. 2011). It is known that HAM/TSP is a late manifestation of HTLV-1 infection occurring usually after the fourth decade of life. However, children who had a previous history of infective dermatitis develop HAM/TSP during adolescence or as young adults (de Oliveira et al. 2005). These children with infective dermatitis without evidence of HAM/TSP present PLV and proinflammatory cytokines as high as patients with HAM/TSP supporting the important role of high HTLV-1 proviral load in early progression of HTLV-1 carriers to HAM/TSP (Nascimento et al. 2009). However, in the present study, HTLV-1 carriers with high PVL, followed for long periods of time, did not develop more clinical or neurological disease than HTLV-1 carriers with low PVL. Variability in the PVL has been observed in other studies during the follow-up of HTLV-1-infected subjects (Demontis et al. 2013; Matsuzaki et al. 2001). However, it cannot be ruled out that in these carriers, host defense mechanisms developed during the course of the viral infection contributed to the decrease in the proviral load and the maintenance of a carrier state.

Previous studies have shown that proinflammatory cytokine levels in serum and supernatants of PBMC are higher in patients with HAM/TSP than in HTLV-1 carriers (Grassi et al. 2011; Matsuzaki et al. 2001; Nagai et al. 1998; Olindo et al. 2005). The proinflammatory cytokines such as IL-1β, IL-6, TNF, CXCL9, CXCL10, and IFN-γ participate in the pathology of several chronic inflammatory diseases including rheumatoid arthritis (Pandya et al. 2017), coronary diseases (Hansson 2005), cerebral malaria (Rudin et al. 1997), tegumentary leishmaniasis (Oliveira et al. 2014), and Chagas diseases (Gomes et al. 2003; Poveda et al. 2014). We have previously shown that patients with HAM/TSP display a decreased ability to down-modulate T cell response (Santos et al. 2006) in comparison with HTLV-1 carriers and that CD8 T cells are the main source of IFN-γ and TNF among T cells in HAM/TSP (Santos et al. 2004). We do not know the mechanism through which HTLV-1 infection induces central nervous system damage, but there is much evidence of the participation of proinflammatory cytokines, as well as CD8 T cell activation in the pathology of infectious diseases. Although baseline production of IFN-γ was higher in cases with high proviral loads than in control groups, both groups produced high amounts of IFN-γ and TNF, indicating that a high production of proinflammatory cytokines may occur even in the absence of neurological disease.

A correlation between proinflammatory and anti-inflammatory cytokines has been observed in subjects with HTLV infection, and there is a direct correlation between IFN-γ and IL-10 as well in TNF and IL-10 in HTLV-1 carriers (Carvalho et al. 2001). In the present study, we evaluated if there was a correlation between the cytokines in carriers with high and low PVL. Interestingly, there was no correlation between IFN-γ and IL-10 in the group with high PVL at entry, but there was a strong direct correlation among these cytokines in the last evaluation. It is possible that the correlation between the anti-inflammatory cytokine IL-10 with the proinflammatory cytokine IFN-γ during the follow-up contributes to prevent the damage caused by exacerbated inflammatory response observed in HTLV-1 infection.

The increase in the frequency of cells expressing TNF is associated with pathology in HTLV-1 infection (Santos et al. 2004; Nakamura et al. 1993; Neco et al. 2017). Moreover, while the frequency of cells expressing TNF and the intensity of expression of TNF was similar in CD4+ T cells from HTLV-1 carriers and patients with HAM/TSP, there was higher frequency of CD8+ T cells expression TNF in HAM/TSP than in HTLV-1 carriers suggesting the participation of TNF in the pathogenesis of HAM/TSP. The decrease in TNF observed in patients during the follow-up may help to avoid tissue damage and may be a marker of protection against HAM/TSP.

An analysis of the correlation between PVL and cytokines in the two groups studied showed different profiles. While low PVL carriers showed a direct correlation between IFN-γ and TNF production and PVL in the last evaluation, in the group of subjects with a high PVL, this correlation was not observed. The existence of a correlation between proviral load and cytokine production was observed in other studies (Lima et al. 2016; Starling et al. 2013). However, the enhancement in the production of proinflammatory cytokines may be due to other factors as the nature of T cell activation and production of other cytokines. This may explain the absence of correlation between PVL and proinflammatory cytokines in the high PVL group.

We recognized that this study has some limitations including the identification of other factors associated with decreased susceptibility to the development of HAM/TSP, the limited follow-up of the carriers, and the lack of evaluation TAX-specific CD8^+^ T cells. The presence of HLA-A * 02 or HLA-Cw * 08 was associated with a lower proviral burden and decreased prevalence of HAM/TSP (Assone et al. 2016), and the clonality of the infected CD4 T cells is a marker of ATL (Kagdi et al. 2018). However, the association of HLA class 1 with decreased susceptibility for HAM/TSP was not documented in other study (Taghaddosi et al. 2013) and oligoclonal proliferation of HTLV infected cells does not account for the development of myelopathy (Nozuma and Jacobson 2019). Indeed, a recent study suggests that the polymorphism of the IFN-γ + 874 T/A gene in carriers of the virus may be associated with progression to symptoms and diseases related to HTLV-1 (Queiroz et al. 2018). Although it cannot be ruled out that if the carriers were followed for a longer period, we would observe clinical and neurological diseases or even the development of HAM/TSP; in the present study, we observed neither the appearance of neurological manifestations nor other diseases associated with HTLV-1 in carriers with high PVL, even followed for more than 10 years.

Our data indicate that a large number of HTLV-1 infected subjects with a high PVL and an exaggerated inflammatory response do not develop neurological manifestations, urinary and erectile dysfunction, nor other diseases associated with HTLV-1. These findings suggest that other factors, in addition to the PVL and the inflammatory response observed in the peripheral blood, are necessary for carriers to develop HTLV-1-associated diseases.
